# Commentary: Effect of Skeletal Muscle Native Tropomyosin on the Interaction of Amoeba Actin with Heavy Meromyosin

**DOI:** 10.3389/fphys.2016.00377

**Published:** 2016-08-31

**Authors:** Joseph M. Chalovich, Dylan Johnson

**Affiliations:** ^1^Department of Biochemistry, Brody School of Medicine at East Carolina UniversityGreenville, NC, USA; ^2^Department of Chemistry, East Carolina UniversityGreenville, NC, USA

**Keywords:** troponin, tropomyosin, cardiomyopathy, troponin T, mutations

Troponin-tropomyosin inhibits skeletal and cardiac muscle contraction at low Ca^2+^. Binding of rigor-type myosin S1 to actin-tropomyosin-troponin, particularly at saturating Ca^2+^, produces activation of myosin ATPase activity in excess of that seen in the absence of the regulatory proteins. The binding energy of S1 can overcome the inhibitory activity of troponin (Bremel et al., 1972) and may allow tropomyosin to move deep into the groove of actin. That particular arrangement of actin, tropomyosin, and troponin is a much better activator of ATP hydrolysis than actin alone. That active configuration of actin was called state 2 in the Hill model (Hill et al., 1980) and later named the M state because of its requirement for tight myosin binding.

Eisenberg and Weihing found evidence that troponin itself can stabilize the active state of actin in the absence of high affinity S1 binding (Eisenberg and Weihing, 1970). They showed that troponin-tropomyosin enhanced the ability of amoeba actin to activate myosin S1 ATPase activity at high Ca^2+^. That observation is often overlooked but may be an important clue to managing some muscle disorders. Actin filaments containing the hypertrophic cardiomyopathy associated Δ14 mutation of TnT also enhanced S1 ATPase rates 2-3-fold higher than actin filaments without bound regulatory proteins (Gafurov et al., 2004). Because small changes in the structure of actin or troponin allow this increased activation to occur, the troponin complex must have a latent ability to enhance actin activation of myosin ATPase activity. The 14 C-terminal residues of TnT attenuate the ability of troponin to enhance actin activation. Troponin containing Δ14 TnT might act by stabilizing tropomyosin in the M state position of the actin groove under saturating Ca^2+^ conditions.

The inactive state of actin-tropomyosin-troponin (state 1 or the B state) occurs at low free Ca^2+^ when the inhibitory region of TnI is bound to actin. Because of associations among the regulatory proteins, tropomyosin is stabilized outside of the actin groove and there is little stimulation of myosin ATPase activity. Removal of the 14 C-terminal residues of TnT prevents formation of the B state. Compared with wild type actin filaments in EGTA, those containing Δ14 TnT exhibit less cooperativity in equilibrium binding of myosin S1 (Gafurov et al., 2004), and they do not exhibit the acrylodan tropomyosin fluorescence increase under conditions favoring the inactive state (Borrego-Diaz and Chalovich, 2010; Franklin et al., 2012).

Ca^2+^ binding to TnC opens a hydrophobic patch to which the switch region of TnI can bind (Herzberg et al., 1986). Under this condition, TnI is detached from actin and tropomyosin is situated in the actin groove. Several lines of evidence indicate that the major state formed with Ca^2+^ is a second inactive state with tropomyosin partially in the actin groove (Trybus and Taylor, 1980; McKillop and Geeves, 1991; Lehman et al., 2001; Kimura et al., 2002; Pirani et al., 2005; Poole et al., 2006). Full movement into the groove to form the active M state requires rigor S1 binding or a structural change in troponin. In the Hill model of regulation, Ca^2+^ binding to troponin was thought to create an inactive state 1 with bound Ca^2+^. State 1 with bound Ca^2+^ may be equivalent to the state intermediate between the B and M states that is called the C state (because of its link to Ca^2+^). The level of activation of ATPase activity at saturating Ca^2+^ is determined by the amount of M state formed in its equilibrium with the C state. The major state formed with Δ14 TnT containing actin filaments at low Ca^2+^ is likely to be the C state as the B state cannot form. The C state is also stabilized by a hypertrophic cardiomyopathy causing mutation, R146G TnI. The R146G mutation in TnI gives relative stabilization to the C state at low Ca^2+^ and the C state is highly stabilized at saturating Ca^2+^(Mathur et al., 2009). An analysis of ATPase rates of R146G TnI containing actin filaments supported the idea that the C state is ineffective in stimulating myosin ATPase activity.

Studying natural mutations and modifications of troponin has given muscle researchers insights into the regulation of contraction. Long term deviations from the normal distribution of B, C, and M states of regulated actin seem to lead to progressive cardiac dysfunction. The last 14 residues of human cardiac TnT are critical for controlling the equilibria among the B, C, and M states of regulated actin; they stabilize the B state at low Ca^2+^ and destabilize the M state at saturating Ca^2+^. Table 1 compares the C-terminal sequences of several forms of troponin T. Note the conservation of the four terminal residues and the pattern of basic residues (bold). The regularly spaced basic residues suggest the possibility of acidic target sites for controlling both the B and M states.

**Table 1:** **C-terminal troponin T sequence comparison**.

Human	Cardiac TNT3	S**K** T**R**G**K** A**K**VT G**R**W**K**
Cow	Cardiac TNNT2	S**K** T**R**G**K** A**K**VT G**R**W**K**
Pig	Cardiac isoform 3	S**K** T**R**G**K** A**K**VT G**R**W**K**
Mouse	Cardiac TNNT2	S**K** T**R**G**K** A**K**VT G**R**W**K**
Human	Fast Skeletal TNN2	AG TPA**K** G**K**VG G**R**W**K**
Human	Slow Skeletal TNN1	R**K** GAG**K** G**R**VG G**R**W**K**
Rabbit	Fast Skeletal TNNT3	AG TTA**K** G**K**VG G**R**W**K**
Chicken	Fast Skeletal TNNT3	**KK** AGA**K** G**K**VG G**R**W**K**

The C-terminal region of TnT might function by directly affecting movement of tropomyosin on the actin surface. The C-terminal region could destabilize the active M state at saturating Ca^2+^ by interfering with tropomyosin movement into the actin groove. At low Ca^2+^, the C-terminal region of TnT could participate in holding tropomyosin away from the actin groove. The C-terminal 14 residues of TnT could also potentially alter the pathway of transmission of information from Ca^2+^ binding to TnC through the events leading to tropomyosin repositioning. Deciphering the mechanisms of action of the C-terminal region of TnT may lead to new therapies for cardiac disorders.

## Author contributions

All authors listed, have made substantial, direct and intellectual contribution to the work, and approved it for publication.

## Funding

Funded by NIH grant AR44504 and the Brody Brothers Grant to JC.

### Conflict of interest statement

The authors declare that the research was conducted in the absence of any commercial or financial relationships that could be construed as a potential conflict of interest.
